# Blunt versus sharp expansion of uterine incision during lower segment cesarean section

**DOI:** 10.1186/s12884-025-08466-3

**Published:** 2025-11-25

**Authors:** Ahmed M. Zeinhom, Sherif M. Habib, Ahmed Eraky Atta, Ahmed M. Essam

**Affiliations:** Obstetrics and Gynecology Department, Faculty of Medicine, Ain Shams University, Cairo, Egypt

**Keywords:** Uterine incision, Cesarean section, Intraoperative hemorrhage

## Abstract

**Background:**

The method of uterine incision expansion throughout lower segment cesarean section (LSCS) may impact intraoperative blood loss and surgical outcomes. This research aims to compare the effects of blunt versus sharp expansion of the uterine incision on intraoperative hemorrhage by assessing changes in hematocrit (HCT) levels. Secondary outcomes include the incidence of uterine artery injury, the need for intraoperative blood transfusion, and the duration of the cesarean section procedure.

**Methods:**

Comparative prospective research was performed at Ain Shams University Maternity Hospital. A total of 120 pregnant women meeting inclusion criteria were randomly assigned into two groups: Group A (blunt expansion using fingers) and Group B (sharp expansion using scissors). Preoperative and postoperative HCT levels were measured to assess intraoperative blood loss. Additional parameters were recorded and analyzed utilizing SPSS software. A p-value < 0.05 was considered significant.

**Results:**

The blunt expansion group demonstrated significantly lower intraoperative blood loss, as indicated by a smaller decline in HCT levels (mean difference: 0.9%) compared to the sharp expansion group. Hemoglobin and red blood cell count also showed significantly better preservation in the blunt method group. There was insignificant variance among the two groups regarding uterine artery injury incidence, the need for blood transfusion, or the duration of the cesarean section procedure.

**Conclusion:**

Blunt expansion of the uterine incision during LSCS was related to reduced intraoperative blood loss. The findings suggested that blunt expansion may be the preferable technique in cesarean deliveries to minimize blood loss while maintaining surgical efficiency.

## Background

Cesarean section (CS) is a globally recognized surgical intervention in childbirth, significantly reducing maternal and fetal morbidity and mortality [1]. Despite its life-saving benefits, CS is associated with several intraoperative and postoperative complications, including excessive blood loss, uterine trauma, and increased surgical time [2].

A key determinant of these outcomes is the technique used for uterine incision expansion. Two widely employed methods—blunt expansion using fingers and sharp expansion using scissors—have been the subject of considerable debate regarding their impact on maternal health [3]. Blunt expansion of the uterine incision involves manually widening the initial incision using fingers, a technique believed to reduce trauma to surrounding tissues and minimize the risk of excessive hemorrhage [4].

Sharp expansion, a surgical technique that uses surgical scissors to control incision length, may offer precision but may increase the risk of vessel injury and blood loss [5]. The global increase in repeat cesarean sections necessitates the identification of the most efficient and safe uterine incision expansion technique for optimal surgical outcomes [6]. Lower segment cesarean section (LSCS) is primarily concerned with intraoperative hemorrhage, which can significantly affect maternal hemodynamic stability and postoperative recovery [7].

Hematocrit level measurement serves as a reliable indicator of blood loss, allowing for an objective comparison between blunt and sharp expansion methods [8]. The advantages and drawbacks of each technique must be comprehensively assessed considering factors like uterine artery injury, intraoperative blood transfusion need, and surgical duration [9].

The study analyzed uterine incision expansion during LSCS, comparing blunt and sharp methods, aiming to improve surgical practices and maternal safety by examining blood loss and secondary measures.

This study evaluates the effectiveness and safety of blunt versus sharp expansion in uterine incision expansion, contributing to the ongoing efforts to minimize surgical risks and enhance recovery for pregnant women undergoing repeated cesarean sections.

This research aimed to fill the knowledge gap and inform evidence-based decisions for obstetricians performing cesarean sections, guiding surgical practice towards the most effective and safe uterine incision expansion technique, improving maternal health outcomes.

## Methods

This six-month prospective comparative study took place at Ain Shams University Maternity Hospital. Before starting, the research protocol received approval from the Faculty of Medicine’s Scientific Research Ethics Committee at Ain Shams University, ensuring adherence to international ethical guidelines such as the Declaration of Helsinki and institutional review board standards. The study was approved by Code number FWA 000017585.

The research enrolled pregnant women between the ages of 18 and 35 who had a BMI ranging from 18 to 34.9 kg/m². Eligible participants had a normal placental insertion, a normal coagulation profile, and normal blood pressure at the time of surgery. Gestational age was limited to 36–40 weeks, and patients included were either primigravida, those with one prior cesarean section (P1CS), or those with two prior cesarean sections (P2CS). Women with a history of abdominal or pelvic surgeries other than cesarean section were excluded, as these could contribute to uterine trauma and increased maternal blood loss. Additionally, patients with multiple pregnancies, fetal macrosomia exceeding 4000 g, anemia with hemoglobin levels below 8.5 g/dL, sepsis, or coagulopathy were also excluded to ensure homogeneity of the study population and to eliminate confounding factors affecting intraoperative blood loss.

Before inclusion in the research, participants received full explanations about the objectives, methodology, risks, and expected benefits of the research. Eligible participants were provided with a patient information sheet outlining the purpose of the research, the nature of the surgical techniques being compared, and the methods of data collection. The information was explained in clear and simple language to ensure full comprehension, and patients were given the opportunity to ask questions and seek clarifications before providing consent.

Involvement in the research was optional, and participants were assured they could leave the research at any point without consequences to their care. Written consent was collected from all subjects before participation, adhering to the ethical protocols of Ain Shams University’s Faculty of Medicine Research Ethics Committee. The consent form included a statement affirming the confidentiality of patient data and ensuring that all information collected would be used solely for research purposes. To protect patient privacy, all information were anonymized and securely stored, accessible only to authorized research personnel.

Strict confidentiality measures were applied to all collected patient data throughout the study to protect participants’ privacy. Each patient was assigned a unique identification code, and no personally identifiable information was included in the research database. Medical records, laboratory results, and surgical details were stored in a secure, password-protected system accessible only to authorized research personnel.

Individuals were randomly allocated to one of two groups. Group A (blunt expansion using fingers) and Group B (sharp expansion using scissors). The primary outcome was the change in hematocrit (HCT) levels preoperatively and postoperatively to assess intra-operative blood loss. Secondary outcomes included the incidence of uterine artery injury, the need for intra-operative blood transfusion, and the duration of the cesarean section procedure. Standardized pre-surgical and surgical protocols were applied to both groups, ensuring uniformity in operative techniques, anesthesia, and postoperative care.

Based on data from Sadaf et al. (2021), which found a smaller hematocrit decline in the blunt expansion group (3.21 ± 1.3) versus the sharp expansion group (4.21 ± 2.17), we calculated the sample size in PASS 15 with 80% power and a 5% alpha error. Using these values and adjusting for a 10% dropout rate, a minimum of 60 patients per group (total 120 patients) will be required [10].

**Fig. 1:**
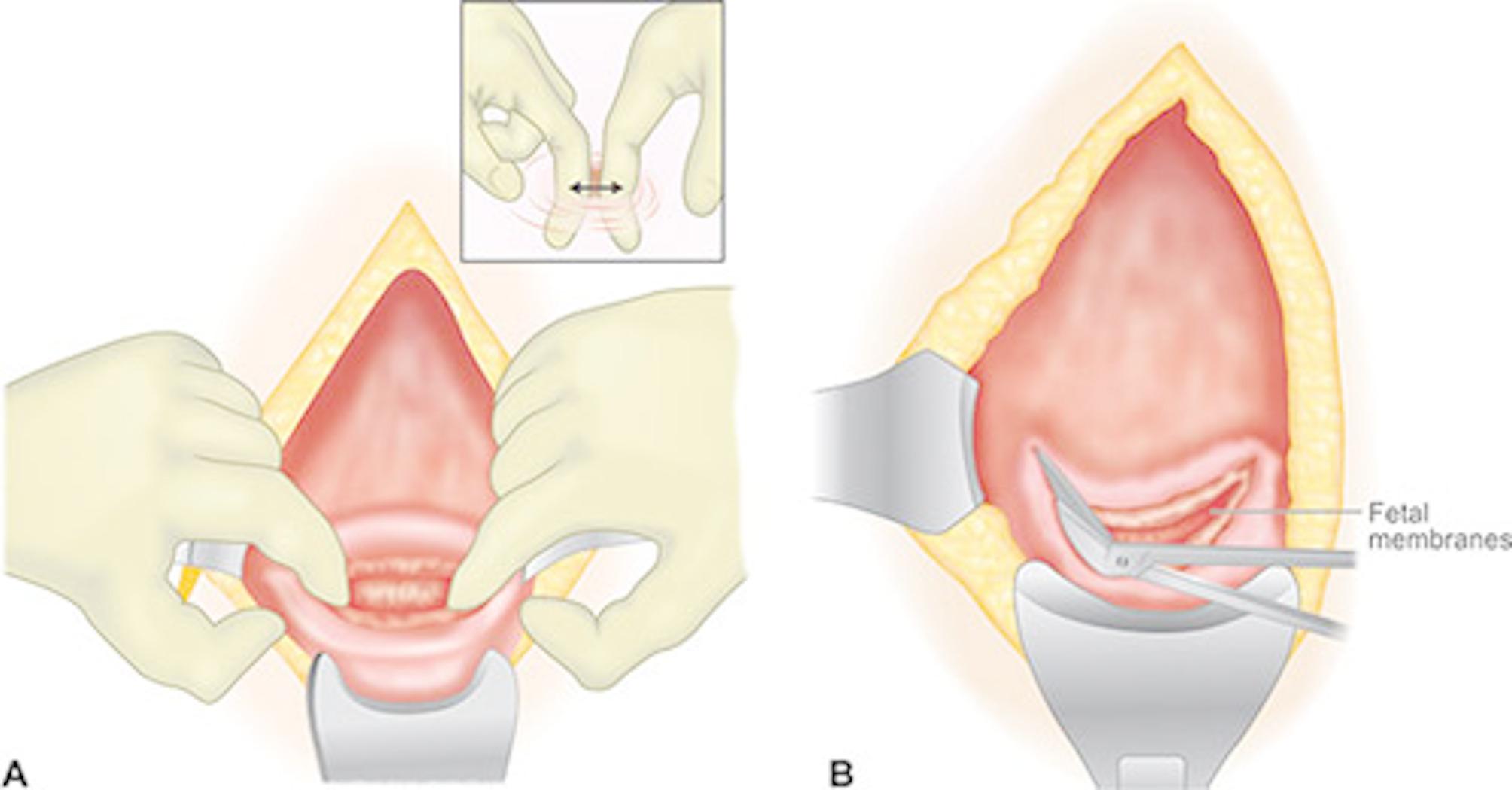
**A** Blunt versus **B** sharp expansion of uterine incision [11]

Patients in this research were randomly divided into the blunt or sharp expansion group via a computer-based randomization method (www.Randomizer.org) (Fig. 1). Allocation concealment was strictly maintained to minimize bias. Before surgery, all participants underwent a thorough preoperative assessment, including demographic, clinical, and laboratory evaluations, which were recorded in standardized case report forms (CRF). Baseline hematocrit (HCT) levels were measured to assess preoperative blood status. Spinal anesthesia was administered using 12 mg of bupivacaine combined with 25 mcg of fentanyl, and prophylactic antibiotics were given before the procedure.

For all participants, a low transverse skin incision (Pfannenstiel incision) was made, followed by a combination of sharp and blunt dissection to access the abdominal cavity. After separating the rectus muscles along the midline, a scalpel was used to create a transverse incision approximately 2 cm in length in the lower uterine segment. The method of uterine incision expansion varied depending on the assigned group: in Group A, expansion was performed bluntly using fingers, while in Group B, it was performed sharply using scissors. After delivery of the fetus and the placenta, a slow intravenous administration of 5 IU oxytocin was given to promote uterine contraction. The uterus was then closed in two layers using polyglactin sutures (Vicryl 0), and the abdominal muscles and rectus sheath were approximated with Vicryl 1 sutures. In cases where the subcutaneous tissue depth was greater than 2 cm, interrupted 2 − 0 Vicryl sutures were applied, and the skin was approximated using a subcuticular 3 − 0 Vicryl stitch.

Postoperative follow-up included an evaluation of intraoperative hemorrhage by measuring changes in hematocrit (HCT) levels before and after the cesarean section. Hematocrit levels were recorded preoperatively and postoperatively to determine the extent of blood loss associated with the two different techniques of uterine incision expansion—blunt expansion using fingers versus sharp expansion using scissors. A significant drop in hematocrit levels was used as an objective indicator of intraoperative bleeding, allowing for a direct comparison between the two methods. The secondary outcomes included several key surgical and clinical parameters. One of the secondary measures was the incidence of uterine artery injury in each group, as this could influence maternal blood loss and surgical complexity. Additionally, the study evaluated the need for intraoperative blood transfusion, which served as an indicator of excessive hemorrhage requiring medical intervention. Another important secondary outcome was the duration of the cesarean section procedure, measured in minutes from the first incision to the completion of suturing. These secondary outcomes provided a broader assessment of the safety, efficiency, and potential complications associated with each method of uterine incision expansion. Data was collected using case report forms (CRF) and entered into Microsoft Excel 2013 for processing. Data were analyzed using SPSS 25. A p-value < 0.05 was considered significant.

## Results

This table presents a comparison between the blunt method (using fingers) and the sharp method (using scissors), focusing on participants age and BMI. The results show insignificant variances among the two groups for both age (*p* = 0.255) and BMI (*p* = 0.172), as both p- values are above 0.05, indicating statistical insignificance. The average age for the blunt method group was 25.5 years, while it was 24.5 years for the sharp method group. The average BMI was 29.9 kg/m² for the blunt group and 28.9 kg/m² for the sharp group. (Table 1)

**Table 1:** Comparison of age and BMI between blunt and sharp methods

		Blunt by fingers (*n* = 60)		Sharp by scissors (*n* = 60)		Test value	P-value
		N	%	N	%		
Age (year)	Mean ± SD	25.5 ± 5.21		24.5 ± 3.75		t = 1.144	0.255
	Range	18–35		18–34			
BMI (Kg/m ^2^)	Mean ± SD	29.9 ± 3.98		28.9 ± 3.76		t = 1.373	0.172
	Range	19–34		22–34			

Using: t-Independent Sample t test for Mean ± SD

X2 = Chi- Square test, p-value > 0.05 is insignificant

*p-value < 0.05 is significant

**p-value < 0.01 is highly significant

This table compares the medical and surgical histories of participants using blunt (fingers) and sharp (scissors) methods. The results show insignificant variances among the two groups in terms of medical history (p=0.545) and surgical history (p=0.552), with both p-values exceeding the threshold for statistical significance (0.05). The majority of participants in both groups were free from medical conditions, with only a small percentage having conditions like bronchial asthma or arthritis. Similarly, surgical histories show that both groups had comparable rates of previous surgeries, including cesarean sections, D&C, tonsillectomy, and Cataract procedures (Table 2).

**Table 2:** Comparison of medical and surgical histories between blunt and sharp methods

		Blunt by fingers (*n* = 60)		Sharp by scissors (*n* = 60)		Test value	P-value
		N	%	N	%		
Medical History	Free	59	98.3	56	93.3	3.078	0.545
	Broncial Asthma	1	1.7	1	1.7		
	D.M.	0	0	1	1.7		
	Rh.A	0	0	2	3.4		
Surgical History	Free	9	15.0	4	6.8	6.855	0.552
	1 Cs	28	46.7	30	50.8		
	2 Cs	11	18.3	14	23.7		
	D&C	5	8.3	5	8.3		
	Others	7	11.7	6	10		

Using: X2 = Chi- Square test, p-value > 0.05 is insignificant

*p-value < 0.05 is significant

**p-value < 0.01 is highly significant

No significant differences were observed in RBC count (p=0.143) and WBC count (p=0.217), with the sharp method group showing a slightly lower RBC count and a higher WBC count. Similarly, hemoglobin (Hb), hematocrit (HCT), platelet count (Plts), clotting times (PT, PTT, INR), liver enzymes (ALT), and creatinine levels didn’t vary significantly among groups (p>0.05) (Table 3).

**Table 3:** Preoperative hematological and biochemical comparisons between blunt and sharp methods

		Blunt by fingers (*n* = 60)	Sharp by scissors (*n* = 60)	Test value	*P*-value
Hb (g/dL)	Mean ± SD	10.9 ± 1.23	10.9 ± 0.87	0.102	0.919
	Range	8.9–13.8	8.8–13.1		
HCT (%)	Mean ± SD	33.5 ± 2.37	32.9 ± 1.36	0.250	0.803
	Range	28.1–41.1	30.7–40.3		
RBCs (10^6/uL)	Mean ± SD	4.1 ± 0.31	4.0 ± 0.17	1.452	0.143
	Range	3.4–4.8	3.67–4.52		
WBCs (10^3/uL)	Mean ± SD	9.4 ± 2.44	10.4 ± 2.55	1.625	0.217
	Range	5.3–14.9	5.3–16.7		
Plts (10^3/uL)	Mean ± SD	233.0 ± 73.1	258.1 ± 79.68	1.796	0.075
	Range	113–438	113–606		
PT (sec)	Mean ± SD	12.0 ± 1.15	11.8 ± 0.94	0.893	0.374
	Range	10.2–14.9	10.3–14.1		
PTT (sec)	Mean ± SD	31.3 ± 2.61	30.6 ± 1.82	1.764	0.080
	Range	26.3–39.0	27.0–33.2		
INR	Mean ± SD	0.98 ± 0.06	0.98 ± 0.07	0.573	0.568
	Range	0.9–1.16	0.9–1.20		
ALT (IU/L)	Mean ± SD	14.9 ± 7.59	13.0 ± 5.35	1.598	0.113
	Range	8–64	7–33		
Creatinine (mg/dL)	Mean ± SD	0.55 ± 0.07	0.54 ± 0.06	0.383	0.703
	Range	0.40–0.72	0.43–0.79		

Using: t-Independent Sample t test for Mean ± SD

p-value > 0.05 is insignificant

*p-value < 0.05 is significant

**p-value < 0.01 is highly significant

The blunt method group showed significantly higher hemoglobin (Hb) levels (10.3 ± 1.11 g/dL) than the sharp method group (9.9 ± 0.90 g/dL) (*p* < 0.001). Hematocrit (HCT) and RBC count were also significantly greater in the blunt group (32.6 ± 2.59% and 3.9 ± 0.33 × 10⁶/uL) than in the sharp group (30.7 ± 2.59% and 3.7 ± 0.11 × 10⁶/uL), with p-values of 0.001 and 0.029, correspondingly. No significant differences were found in WBC count, platelet count, or coagulation parameters (*p* > 0.05). (Table 4)

**Table 4:** Postoperative hematological and biochemical outcomes between blunt and sharp methods

		Blunt by fingers (*n* = 60)	Sharp by scissors (*n* = 60)	Test value	*P*-value
Hb (g/dL)	Mean ± SD	10.3 ± 1.11	9.9 ± 0.90	4.036	< 0.001**
	Range	7.7–12.3	8.1–13.1		
HCT (%)	Mean ± SD	32.6 ± 2.59	30.7 ± 2.59	3.291	0.001**
	Range	23.7–38.1	22.7–37.1		
RBCs (10^6/uL)	Mean ± SD	3.9 ± 0.33	3.7 ± 0.11	2.216	0.029*
	Range	2.9–4.5	3.5–4.1		
WBCs (10^3/uL)	Mean ± SD	12.5 ± 2.52	13.2 ± 2.21	2.425	0.125
	Range	6.9–16.4	8.6–17.5		
Plts (10^3/uL)	Mean ± SD	213.4 ± 61.48	230.9 ± 75.98	1.390	0.167
	Range	101–394	98–585		
PT (sec)	Mean ± SD	13.4 ± 1.0	13.9 ± 0.83	0.380	0.704
	Range	11.3–15.4	12.2–15.9		
PTT (sec)	Mean ± SD	33.1 ± 2.59	32.7 ± 1.81	0.884	0.379
	Range	27.8–40.0	29.4 − 35.3		
INR	Mean ± SD	1.05 ± 0.05	1.08 ± 0.04	0.153	0.879
	Range	1.0–1.18	1.01–1.20		

Using: t-Independent Sample t test for Mean ± SD

p-value > 0.05 is insignificant

*p-value < 0.05 is significant

**p-value < 0.01 is highly significant

The sharp method group showed a notably larger decline in Hb, HCT, and RBC count than the blunt method group. Hb levels dropped by an average of 0.6 g/dL (p<0.001) with the blunt technique and 1.0 g/dL (p<0.001) with the sharp technique. HCT declined by 0.9% (p=0.003) with the blunt method and 2.2% (p<0.001) with the sharp method. RBC count dropped by 0.2 ×10⁶/uL (p=0.006) in the blunt group and 0.3 ×10⁶/uL (p<0.001) in the sharp group (Table 5).

**Table 5:** Mean difference between preoperative and postoperative hematological parameters for blunt and sharp methods

Mean Difference		Blunt by fingers (*n* = 60)	Sharp by scissors (*n* = 60)	Test value	*P*-value
Hb	Mean ± SD	−0.6 ± 1.65	−1.0 ± 1.25	4.615	< 0.001**
	Range	−6.1–3.4	−4.3–4.3		
HCT	Mean ± SD	−0.9 ± 1.51	−2.2 ± 2.77	3.030	0.003**
	Range	−1.7–1.0	−4.6–1.0		
RBCs	Mean ± SD	−0.2 ± 0.51	−0.3 ± 0.87	2.817	0.006**
	Range	−0.7–0.4	−1–1.1		

Using: t-Independent Sample t test for Mean ± SD

p-value > 0.05 is insignificant

*p-value < 0.05 is significant;

**p-value < 0.01 is highly significant

Uterine artery injury and the need for ligation occurred in 3.3% of cases in the sharp method group but were absent in the blunt method group. Nevertheless, this variance wasn’t statistically significant (p=0.154). Both groups had no cases requiring blood transfusion, suggesting that the choice of method did not impact intraoperative blood loss significantly (Table 6).

**Table 6:** Intraoperative comparison of uterine artery injury and blood transfusion between blunt and sharp methods

		Blunt by fingers (*n* = 60)		Sharp by scissors (*n* = 60)		Test value	P-value
		N	%	N	%		
Uterine artery injury	Yes	0	0	2	3.3	2.034	0.154
	No	60	100	58	96.7		
Need for ligation of uterine artery	Yes	0	0	2	3.3	2.034	0.154
	No	60	100	58	96.7		
Need for Blood transfusion	Yes	0	0	0	0	-	-
	No	60	100	60	100		

Using: X2 = Chi- Square test, p-value > 0.05 is insignificant

*p-value < 0.05 is significant

**p-value < 0.01 is highly significant

The mean duration for the blunt method was 46.4± 6.04 minutes, while the sharp method took slightly longer at 47.8 ± 5.77 minutes. Nevertheless, this variance lacked statistical significance (p=0.192), suggesting that the surgical approach did not substantially influence the total procedure time (Table 7).

**Table 7:** Comparison of cesarean section duration between blunt and sharp methods

		Blunt by fingers (*n* = 60)	Sharp by scissors (*n* = 60)	Test value	*P*-value
C.S duration (in minutes)	Mean ± SD	46.4 ± 6.04	47.8 ± 5.77	1.313	0.192
	Range	35–55	35–60		

Using: t-Independent Sample t test for Mean ± SD

p-value > 0.05 is insignificant

*p-value < 0.05 is significant

**p-value < 0.01 is highly significant

## Discussion

A key strength of this study is the comparable baseline characteristics of the two groups, ensuring a robust comparison of the surgical techniques. Demographic factors, such as age and BMI, showed no significant differences between the groups, with mean values falling within a narrow range. Additionally, the absence of significant variations in medical and surgical histories, including rates of prior cesarean deliveries and other procedures, further underscores the comparability of the study populations. These balanced baseline conditions are crucial for isolating the impact of the surgical techniques on the outcomes.

In the same line Shaheen et al. [12] reported no significant differences in demographic or clinical variables such as age, BMI, and parity distribution between blunt and sharp groups, supporting the consistency of randomization in comparative studies. Similarly,Morales et al. [13]. emphasized the importance of balanced preoperative characteristics, including gestational age and parity, in ensuring that any observed differences in outcomes could be directly attributed to the surgical techniques rather than inherent baseline disparities. In contrast, El-Berry et al. [14]., identified slight variations in BMI and parity between groups, which could potentially affect intraoperative outcomes. Despite these minor discrepancies across studies, the consistent use of randomization and methodological rigor strengthens the reliability of findings and highlights the importance of achieving balanced baseline conditions to isolate the true impact of uterine incision expansion techniques on maternal and fetal outcomes.

Intraoperative safety was a central focus of this study. Notably, no uterine artery injuries occurred in the blunt group, while two cases (3.3%) were recorded in the sharp group. Although the variance wasn’t statistically significant, the absence of injuries in the blunt group supports its potential as a safer technique. Moreover, neither group required intraoperative blood transfusions, indicating that both methods are associated with minimal perioperative risks in this regard. These findings align with previous research suggesting that blunt expansion may impose less vascular stress on the uterine tissues, potentially reducing the risk of inadvertent injuries.

Intraoperative safety, as highlighted in this study, aligns with findings from prior research, though some variations exist. The absence of uterine artery injuries in the blunt group and the minimal perioperative risks observed are consistent with findings from Morales et al. [13] who reported that blunt uterine expansion techniques were associated with a lower risk of surgical complications, including vessel injury and unintended extensions, compared to sharp methods. Similarly, Sadaf et al. [10] observed significantly reduced blood loss and fewer complications with blunt expansion, attributing this to decreased vascular stress on uterine tissues.

One of the most striking differences observed was in hematological parameters, where the blunt method consistently outperformed the sharp method. Postoperative hemoglobin levels were significantly higher in the blunt group (10.3 ± 1.11 g/dL) compared to the sharp group (9.9 ± 0.90 g/dL, P < 0.001, P < 0.001). Similarly, postoperative hematocrit levels were better preserved in the blunt group (32.6 ± 2.59% vs. 30.7 ± 2.59%, P = 0.001P = 0.001). Red blood cell counts also showed a smaller decline in the blunt group (− 0.2 ± 0.51 − 0.2 ± 0.51) compared to the sharp group (− 0.3 ± 0.87 − 0.3 ± 0.87, P = 0.006, P = 0.006). The mean differences between preoperative and postoperative values further emphasize the advantage of the blunt method in minimizing blood loss. These findings are consistent with prior studies, which have suggested that blunt expansion may reduce bleeding at the incision site due to less disruption of vascular structures.

The observed advantage of the blunt method in preserving hematological parameters aligns with findings from several studies, although some variations exist. Morales et al. [13] reported that blunt uterine expansion techniques were associated with lower hemoglobin declines and reduced risk of excessive bleeding compared to sharp methods, attributing this to minimized vascular disruption. Similarly, Sadaf et al. [10] found significantly less reduction in hemoglobin (1.47 ± 1.08 g/dL vs. 1.95 ± 0.85 g/dL, P = 0.031P = 0.031) and hematocrit levels in the blunt group, reinforcing the blunt method’s effectiveness in controlling intraoperative blood loss.

However, El-Berry et al. [14] observed that while blunt methods generally reduced blood loss, the differences in hematological outcomes between the two groups were less pronounced, and sharp methods occasionally demonstrated faster healing times, highlighting that the clinical superiority of blunt methods may depend on context and execution.

Despite the differences in hematological outcomes, the duration of cesarean sections was comparable between the groups. The blunt method required 46.4 ± 6.04 min, slightly shorter than the 47.8 ± 5.77 min for the sharp method, but the variance wasn’t statistically significant (P = 0.192P = 0.192). This indicates that the choice of technique does not significantly affect operative efficiency, making the blunt method a practical choice for clinicians aiming to optimize maternal outcomes without extending surgical time.

The comparable operative durations observed in this study are consistent with findings from prior research, though some studies report slight variations. Morales et al. [13] noted no significant difference in surgical duration between blunt and sharp uterine expansion methods, attributing this to the standardized approach to both techniques in their trial. Similarly, Sadaf et al. [10] reported that both methods required comparable time to perform, reinforcing the idea that the choice of technique does not compromise surgical efficiency.

However, El-Berry et al. [14] suggested that sharp methods might offer a slight edge in speed due to the controlled and precise nature of the incision expansion, though they acknowledged that the difference was not clinically significant. These findings collectively suggest that while operative time remains unaffected by the choice of technique in most cases, individual surgeon preferences and experience may play a role in determining efficiency.

## Conclusion

This research revealed that expanding the uterine incision using the blunt technique maintains hematological levels more effectively than the sharp method, suggesting less blood loss. Operative duration, uterine artery injury rates, and blood transfusion needs were comparable, suggesting similar safety profiles. These results highlight the blunt method’s potential advantage in minimizing blood loss without compromising surgical efficiency or safety, making it a preferable technique for cesarean deliveries.

## Data Availability

Upon reasonable request, the corresponding author can provide access to the datasets used and/or analyzed in this study.

## Abbreviations

CS: Cesarean section
HCT: Hematocrit
Hb: Hemoglobin
INR: International normalized ratio
P1CS: One prior cesarean section
P2CS: Two prior cesarean sections
CRF: Case report form
